# Safety and protective effects of maternal influenza vaccination on pregnancy and birth outcomes: A prospective cohort study

**DOI:** 10.1016/j.eclinm.2020.100522

**Published:** 2020-09-09

**Authors:** Hassen Mohammed, Claire T. Roberts, Luke E. Grzeskowiak, Lynne C. Giles, Gustaaf A. Dekker, Helen S. Marshall

**Affiliations:** a Vaccinology and Immunology Research Trials Unit, Women's and Children's Health Network, Adelaide, South Australia, Australia; b Robinson Research Institute, The University of Adelaide, Adelaide, South Australia, Australia; c Adelaide Medical School, The University of Adelaide, Adelaide, South Australia, Australia; d College of Medicine and Public Health, Flinders University, Bedford Park, South Australia, Australia; e SA Pharmacy, Flinders Medical Centre, SA Health, Bedford Park, Adelaide, Australia; f School of Public Health, The University of Adelaide, Adelaide, South Australia, Australia; g Women's and Children's Division, Lyell McEwin Hospital, Elizabeth Vale, South Australia, Australia

## Abstract

**Background:**

Our study aimed to assess the safety and protective effect of maternal influenza vaccination on pregnancy and birth outcomes.

**Methods:**

The study population comprised 1253 healthy nulliparous pregnant women in South Australia between 2015 and 2018. Participants were followed prospectively, with vaccination status (confirmed by medical records), pregnancy, and birth outcome data collected by midwives. Adjusted relative risks (aRRs) and adjusted hazard ratios (aHRs) were estimated accounting for time-varying vaccine exposure and temporal nature of each outcome.

**Findings:**

Maternal influenza vaccination (48%, 603 of 1253) reduced the risk for pre-delivery hospitalisation with influenza like illness (aHR 0•61; 95% CI 0•39, 0•97). Maternal influenza vaccination was not associated with spontaneous abortion (aHR 0•42, 95% CI 0•12, 1•45), chorioamnionitis (aRR 0•78, 95% CI, 0•32, 1•88), gestational hypertension (aHR 0•78, 95% CI 0•47, 1•29), pre-eclampsia (aHR 0.84, 95% CI 0•54, 1•27), gestational diabetes (aHR 1•16, 95% CI 0•82, 1•66) nor preterm birth (aHR 0•94, 95% CI 0•59, 1•49). No associations between antenatal influenza vaccination and congenital anomalies, admission to the neonatal care unit, low Apgar scores, and mechanical ventilation were observed. Results were not materially changed after adjustment for pertussis vaccination. We observed a protective effect of maternal influenza vaccination on low birth weight (aHR 0•46, 95% CI 0•23, 0•94) and a marginal protective effect on small for gestational age births (aHR 0•65, 95% CI 0•40, 1•04) during periods of high influenza activity.

**Interpretation:**

These results support the safety of maternal influenza vaccination and suggest a protective effect in reducing the rates of low birthweight and small for gestational age births.

**Funding:**

There was no funding for this study.

**Research in context panel**

**Evidence before this study**

We searched PubMed for English language studies published until March 31, 2020, with no start date restriction, with the terms “influenza”, “influenza vaccine”, “maternal influenza vaccination”, maternal influenza immunisation”, “humans” and “pregnancy”. The World Health Organization (WHO) considers pregnant women as a priority group for seasonal inactivated influenza vaccination due to their vulnerability to influenza infection and its resulting morbidities. Previous studies have shown that inactivated influenza vaccine during pregnancy is safe and provides passive antibodies to the infant, as well as clinical protection for both mother and infant < 6 months of age against influenza infections and influenza-related hospitalisations. Despite the recommendation of maternal influenza vaccination from immunisation advisory groups internationally including WHO, it has not been implemented in most low-resource countries, and even in high income countries where it is incorporated into standard antenatal care, vaccination uptake is often suboptimal. While the body of literature regarding the safety of influenza vaccination during pregnancy is mounting, there are relatively few prospectively designed or clinical trials that include pregnant women. In high income countries where maternal influenza vaccination is recommended, prospectively designed studies with advanced statistical approaches are likely to be the only way to comprehensively assess the safety of influenza immunisation during pregnancy and its important potential protective effects in reducing low birth weight, small for gestational age birth and preterm birth, for which evidence is conflicting.

**Added value of this study**

This prospective cohort of healthy pregnant women, with confirmed vaccination status and accurate pregnancy and infant outcome data used robust nuanced time-to-event analyses. The study showed that influenza vaccination during pregnancy is not associated with adverse pregnancy, foetal or birth outcomes. This study also presents evidence that inactivated influenza vaccination decreases the risk of pre-delivery hospitalisation with maternal influenza-like illness by 39% and reduces the risk of low birthweight and small for gestational age births during periods of high influenza activity.

**Implications of all the available evidence**

These findings provide further reassurance to women and health care providers about the safety of inactivated influenza vaccination during pregnancy. Importantly, our results provide evidence in support of maternal influenza vaccination **reducing** low birth weight and small for gestational age births during periods of widespread influenza activity. These findings need to be replicated in other countries as it is plausible that the impact of maternal influenza vaccine on these birth outcomes may vary with the underlying local influenza epidemiology and demographic characteristics. Our findings could be pivotal for countries weighing the additional benefits of implementing maternal influenza immunisation programs. This may be particularly important for low income countries where the rates of low birthweight and small for gestational age births are very high, and known to be strong risk factors for neonatal and early childhood morbidity and in which health systems have poor capacity to mitigate short and long-term effects.

Alt-text: Unlabelled box

## 1 Introduction

Pregnant women are vulnerable to serious complications from influenza including preterm labour, pneumonia, hospitalisation and death, particularly during seasonal and pandemic influenza outbreaks [1,2]. Newborns whose mothers had influenza during pregnancy are also at increased risk of adverse outcomes such as preterm birth and low birthweight [3,4]. Maternal influenza vaccination protects mothers against influenza infection and their offspring by transplacental antibody transfer from mother to foetus conferring passive immunity until the first influenza vaccination from age six months [5,6]. Influenza vaccination during pregnancy might also reduce the risk of low birthweight, preterm birth, and stillbirth but evidence concerning these birth outcomes is conflicting [7], [8], [9], [10], [11], [12], [13]. Despite recommendations for maternal influenza vaccination, uptake during pregnancy remains suboptimal globally [14].

A major challenge for achieving high uptake of influenza vaccination during pregnancy relates to relatively limited published evidence of vaccine safety for pregnant women and their foetus [8], [9], [10], [11], [12]. A review of factors influencing acceptance of antenatal vaccination indicated that access issues and safety concerns are major barriers to uptake [15]. An inflammatory response from infection during pregnancy has been shown to increase the risk of foetal injury [16] but no evidence exists that an inflammatory response from a vaccine carries a similar risk. A robust assessment of the safety of influenza vaccination during pregnancy is critical due to population-wide rollouts of vaccines for this group. A number of systematic reviews have reported pregnancy and birth safety outcomes following influenza vaccination in pregnancy [8], [9], [10], [11], [12], [13].

Most observational research into vaccine safety and efficacy during pregnancy has been retrospective, due to the relatively cheaper cost, fewer ethical concerns, and difficulty in recruiting pregnant women to randomized controlled trials (RCTs). Whilst providing timely reporting, this approach has limitations. In most retrospective studies, authors have been unable to establish if a pregnancy complication preceded vaccination nor account for the time-dependant nature of exposure to vaccination during pregnancy. In countries where maternal influenza vaccination is recommended, prospectively designed studies are likely to be the only way to accurately determine the true risk or potential benefits of maternal vaccination beyond prevention of influenza for pregnant women and their infants. Our study aimed to prospectively assess maternal and birth outcomes following inactivated influenza vaccination during pregnancy, while also taking into account the most comprehensive set of potential confounding variables considered to date.

## 2 Methods

### 2.1 Study design and participants

The current study draws on data collected as part of a prospective cohort study (STOP), which aims to develop screening tests to identify adverse pregnancy outcomes. Healthy nulliparous women were recruited in pregnancy at two major maternity hospitals, the Lyell McEwin Hospital, the tertiary hospital serving the low socio-economic community in Adelaide's Northern suburbs and the Women's and Children's Hospital, the primary tertiary maternity hospital for complex care, accounting for around 50% of the 16,000 annual births in metropolitan Adelaide, South Australia. Between March 2015 and December 2017, nulliparous women with a singleton pregnancy attending their first antenatal clinic between 9 ^+^ ^0^ and 16^+0^ weeks’ gestation were enroled. Women were excluded if they were considered already at high risk of pregnancy complications at screening (i.e. experienced three or more previous miscarriages or with pre-existing hypertension or diabetes). Participants were followed prospectively, with vaccination, pregnancy, and birth outcome data collected by research midwives. Written informed consent was obtained from all participants included in the STOP study. The original STOP study protocol was approved by the Human Research Ethics Committee of the Women's and Children's Hospital Adelaide Australia (HREC/14/WCHN/90), registered at Australian New Zealand Clinical Trials Registry, ACTRN12614000985684.

### 2.2 Exposure

The exposure of interest was trivalent inactivated influenza vaccination during pregnancy, defined as a vaccine received between the first day (date) of the last menstrual period and the end of pregnancy. A research midwife interviewed and collected maternal vaccination status of the women during their first study visit at 9–16 weeks’ gestation and during their second study visit interview at 32–36 weeks’ gestation. Vaccination date and gestation of administration were recorded. Following delivery, a research midwife interviewed the participants and verified final vaccination status by reviewing medical case notes and Pregnancy-Hand-Held-Record to confirm the reported vaccination status. Pregnancy-Hand-Held-Records are the main medical record of pregnancy care in South Australia and are reviewed and updated at antenatal appointments.

### 2.3 Outcomes

Pregnancy outcomes assessed were pre-delivery admission due to influenza-like illness, spontaneous abortion after inclusion in the STOP study, gestational diabetes, gestational hypertension, pre-eclampsia, severe pre-eclampsia, chorioamnionitis, premature rupture of membranes, spontaneous preterm birth, preterm birth and stillbirth. Birth outcomes included congenital anomalies, small for gestational age (SGA), low birthweight (< 2500 g) (LBW), low birthweight at term (≥ 37 weeks’ gestation), Apgar scores at 1 and 5 min, neonatal care unit admissions, respiratory distress and mechanical ventilation.

Pregnancy and birth complications were diagnosed using the Brighton Collaboration consensus list of terms, and international guidelines. Gestational hypertension was defined as (peripheral) hypertension [systolic BP (SBP) ≥ 140 mmHg or diastolic BP (DBP) ≥ 90 mmHg] after 20 weeks of gestation in previously normotensive women. Pre-eclampsia was defined as gestational hypertension with proteinuria (24 h urinary protein ≥ 300 mg or spot urine protein: creatinine ratio ≥ 30 mg/mmol creatinine or urine dipstick protein ≥ 2+) or any multi-organ complication of pre-eclampsia. Severe pre-eclampsia was defined as pre-eclampsia with one or more of the following clinical features: BP of ≥ 160/110 mmHg or hypertension requiring intravenous therapy with an antihypertensive agent or magnesium sulphate after 20 weeks of gestation. Preterm birth was defined as any birth before 37 and after 20 completed weeks of gestation. SGA was defined as neonates with a birthweight below the <10th percentile customized for maternal factors such as maternal height, booking weight, ethnicity and gestational age at delivery. The estimated date of delivery was calculated from a certain last menstrual period (LMP) date and was only adjusted if either (1) a scan performed at < 16 weeks of gestation found a difference of ≥7 days between the scan gestation and that calculated by the LMP or (2) on 20-week scan a difference of ≥ 10 days was found between the scan gestation and that calculated from the LMP. If the LMP date was uncertain, then scan dates were used to calculate the estimated date of delivery.

### 2.4 Covariates

During the first study visit at 9–16 weeks’ gestation, information was obtained regarding baseline socio-demographic, lifestyle and clinical characteristics such as age, ethnicity, level of education, household income, employment, exercise, smoking, supplement use, intake of alcohol and recreational drugs, medical and obstetric history, and complications during the current pregnancy. Participating women also completed the Perceived Stress Scale (PSS-10), to assess perceived stress levels in the past month, the short form of the Spielberger State–Trait Anxiety Inventory (STAI), assessing current anxiety symptoms, and the Edinburgh Postnatal Depression Scale (EPDS), assessing depressive symptoms during pregnancy.

### 2.5 Statistical methods

Demographic, lifestyle and clinical characteristics of participants were summarized descriptively, by influenza vaccination exposure during pregnancy. Continuous variables were summarized as mean with standard deviation (SD) or median with interquartile range (IQR), as appropriate, while counts and percentages were used to summarize categorical variables. To investigate if there was an association between influenza vaccination status and each of the outcome variables, we initially conducted independent samples t-tests or Mann-Whitney U tests, as appropriate, for continuous variables and chi-square tests of association for binary and categorical variables.

The timing for vaccination exposures and time at risk windows were calculated for each time sensitive pregnancy and birth outcome accounting for the temporal nature of each outcome of interest. For example, women were at risk for preterm birth from 20 weeks until 36^+6^ weeks of gestation. Cox proportional-hazards models with gestational age in weeks as the underlying time metric were used to derive hazard ratios (HRs) that compared the hazard rates for time-sensitive outcomes such as spontaneous abortion or preterm birth between vaccinated and unvaccinated women. Vaccination status was treated as a time-varying exposure in these models, in that each vaccinated woman's pregnancy was decomposed into an unvaccinated exposure period and a vaccinated exposure period. In sensitivity analyses, we estimated HRs and adjusted HRs of time-dependant pregnancy or birth outcomes by trimester of influenza vaccination during pregnancy. To assess the impact of the intensity of influenza activity on the association between maternal influenza vaccination and key birth outcomes, we also stratified analyses by the level of influenza activity at time of delivery using the South Australian Influenza Surveillance Report [17] based on the percentage of laboratory confirmed influenza during the study period 2015–2018. We identified high activity periods as having rates of laboratory confirmed influenza of at least 10% for at least 3 of 4 consecutive weeks. Low influenza activity period was defined as the first week during which the positive rate was lower than 10% and remained at that level for at least four consecutive weeks. On this basis, “high influenza activity” periods were identified for 01 June – 31 October 2015, 01 July – 31 December 2016, 01 June – 30 November 2017 and 31 August – 31 October 2018. The delivery months of the vaccinated women were classified into “high” and “low” influenza activity to compare key birth outcomes of infants born to vaccinated mothers during high/low influenza activity with births occurring at any time to unvaccinated women.

We used log-binomial models to estimate risk ratios (RR) and adjusted risk ratios (aRR) comparing risk of late onset or early postpartum adverse pregnancy outcomes and adverse birth outcomes including congenital anomalies, low Apgar score, admission to neonatal unit, respiratory distress syndrome and mechanical ventilation in infants of vaccinated and unvaccinated mothers. Finally, we used a multivariable linear regression model to predict the difference in mean gestational age at delivery and mean birthweight by vaccination status. For all multivariable (i.e. adjusted) models, annual household income, level of education, ethnicity, maternal health risk factors (age, gravidity, alcohol intake, recreational drug use, smoking, pre-pregnancy body mass index (BMI)), use of micronutrient supplements, asthma and current psychological states were amongst the variables selected as potential confounders based on evidence in the literature [8], [9], [10], [11] guided by directed acyclic graphs. Additional sensitivity analyses were conducted in all multivariable models to evaluate whether the effects of maternal influenza vaccination on pregnancy and birth outcomes were maintained after adjustment for pertussis vaccination in third trimester. As pertussis vaccination was also recommended in pregnancy from 28 to 32 weeks’ gestation in Australia, in our linear regression analyses, we restricted the cohort to women whose pregnancies reached at least 32 weeks’ gestation to allow for all women to have had the opportunity to receive the pertussis vaccine. Missing covariate values are reported in the baseline table where relevant. The amount of missing data is minimal ranging between 0•1% (estimated season of delivery data) to 2•3% (STAI data), and therefore all available data were used in the analyses of all pre-specified outcomes. For all analyses, a p value < 0•05 was considered statistically significant. Data were recorded in a REDCap [18, 19] online database and all statistical analyses were conducted using Stata version 15 (Stata Corp, College Station, Texas, USA).

Role of Funding Source: Not applicable

## 3 Results

**Fig. 1:**
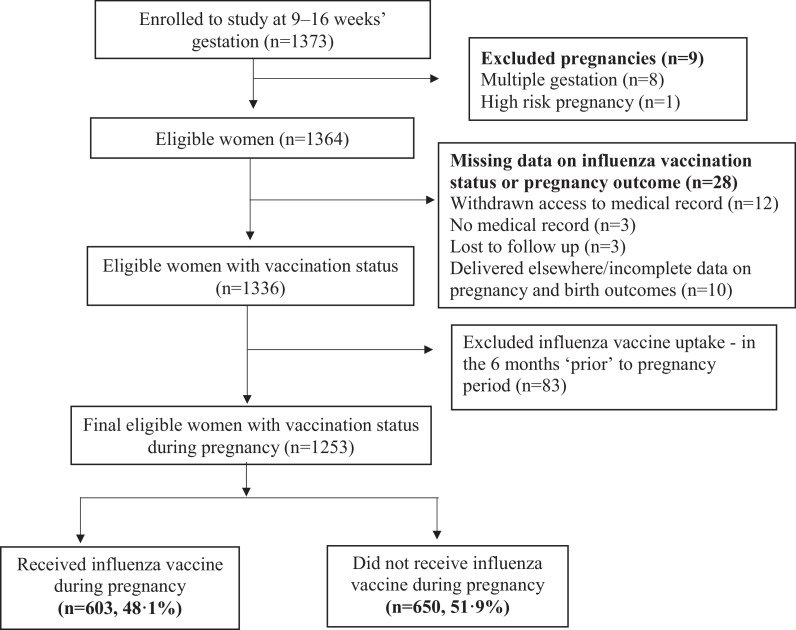
Participants flow diagram.

Of 1364 pregnant women enroled, 12 withdrew access to their medical records, three had no medical case notes and three were lost to follow up or delivered elsewhere (*n* = 10); all 28 were excluded from our final analyses. So as not to confound any observed associations, we excluded 83 women who had influenza vaccination prior to pregnancy. Our final cohort consisted of 1253 women (Fig. 1). Key variables of interest did not differ between women included and excluded from our study (supplementary material p 1). At recruitment, mean maternal age of nulliparous women was 25•9 years (SD 5•0) (range: 15–45 years) and median gestational age was 11•4 weeks (IQR 9•1–12•8) with 82•2% (1031 of 1253) presenting for their first antenatal care visit in the first trimester of pregnancy.

The overall uptake of influenza vaccination was 48•1% (603 of 1253); of the vaccinated women, 24•0% (*n* = 145) were vaccinated in first trimester, 20•2% (*n* = 122) in second trimester, and 55•7% (*n* = 336) in third trimester. Both influenza and pertussis vaccinations occurred in 555 of 1253 (44•2%) pregnancies. Unvaccinated women were more likely to be younger, Aboriginal and/or Torres Strait Islander, in lowest household income group, smoke cigarettes, use illicit drugs, physically inactive, have lower educational attainment and less likely to take micronutrient supplements pre-conception or during pregnancy, and give birth during Autumn compared with vaccinated pregnant women (Table 1).

**Table 1:** Maternal characteristics of vaccinated and unvaccinated pregnant women who delivered at two obstetric hospitals in South Australia, 2015 to 2018 (*N* = 1253).

Variable	Vaccinated women *N* = 603, n (%)	Unvaccinated women *N* = 650, n (%)
Maternal age (years) 15–19 20–24 25–29 >30	50 (8•2) 157 (26•0) 233 (38•6) 163 (27•0)	76 (11•6) 232 (35•6) 212 (32•6) 130 (20•0)
Race/ethnicity Caucasian Aboriginal/TSI Others	492 (81•5) 7 (1•1) 104 (17•2)	542 (83•3) 16 (2•4) 92 (14•1)
Household annual income in AUD <40,000 40,001–70,000 70,001–105,000 >105,001 *Missing*	118 (19•5) 125 (20•7) 165 (27•3) 190 (31•5) 5 (0•8)	184 (28•3) 157 (24•1) 153 (23•5) 146 (22•4) 10 (1•5)
Maternal education ≤ Secondary school qualification Diploma/certificate Bachelor's or higher degree *Missing*	245 (40•6) 215 (35•7) 142 (23•5) 1 (0•1)	311 (47•8) 234 (36•0) 102 (15•6) 3 (0•4)
Smoking status at 9–16 weeks’ gestation Current smoker Quit during pregnancy Non-smoker *Missing*	43 (7•1) 67 (11•1) 491 (81•4) 2 (0•3)	78 (12•0) 71 (10•9) 495 (76•1) 6 (0•9)
Illicit drug use during 1st trimester/pre-pregnancy	29 (4•8)	46 (7•0)
Multivitamin and mineral supplements use Pre-conception and 1st trimester 1st trimester None *Missing*	157 (26•0) 326 (54•0) 116 (19•2) 4 (0•6)	116 (17•8) 345 (53•0) 186 (28•6) 3 (0•4)
Moderate exercise during 1st trimester/pre-pregnancy ≥ 4 per week 1–3 per week Never *Missing*	82 (13•6) 281 (46•6) 237 (39•3) 3 (0•5)	68 (10•4) 300 (46•1) 276 (42•4) 6 (0•9)
Gravidity >1	165 (27•3)	185 (28•4)
Pre-pregnancy asthma	78 (12•9)	90 (13•8)
Pre-pregnancy BMI (kg/m2) <18•5 (Under) 18•5–24 (Normal) 25–29•9 (Overweight) ≥ 30 (Obese)	11 (1•8) 237 (39•3) 168 (27•8) 187 (31•0)	15 (2•1) 255 (39•2) 181 (27•8) 199 (30•6)
Psychological measures at 9–16 weeks’ gestation Edinburgh Perinatal Depression Scale (EPDS) scores, mean (SD) State and trait anxiety (STAI) scores, mean (SD) Perceived stress scale (PSS) scores, mean (SD *Missing*	5•5 ± 4•4 33•4 ± 11•0 13•0 ± 6•4 19 (1.5)	5•4 ± 4•6 33•5 ± 11•1 12•9 ± 6•6 19 (1.5)
Influenza vaccine timing 1st trimester 2nd trimester 3rd trimester	145 (24•0) 122 (20•2) 336 (55•7)	NA
Gestational week of vaccine administration, mean (SD)	23•0 ± 10•5	NA
Received pertussis vaccination during pregnancy	555 (92•0)	398 (61•2)
Estimated season of delivery Summer Autumn Winter Spring *Missing*	171 (28•3) 82 (13•6) 184 (30•5) 166 (27•5) 0 (0)	163 (25•0) 224 (34•4) 135 (20•7) 127 (19•5) 1 (0•1)

Data are mean (SD) or n (%). SD= standard deviations. AUD=Australian dollars. BMI=body-mass-index.

### 3.1 Pregnancy outcomes

Of the 1253 women, 34 (2•7) had spontaneous abortions < 20 weeks’ gestation, seven had terminations (0•5%), six had stillbirths (0•4%), and 1201 (95•8%) delivered a live infant (five missing values). The mean gestational age at delivery was 39•2 weeks (SD 2•0 weeks). Overall, 95 of 1253 (7•5%) women were admitted to hospital due to influenza like illness during pregnancy; mostly in the third (93 of 95) trimesters of pregnancy. The time-dependant Cox proportional hazards regression model shows that women vaccinated at any time during pregnancy had a significant lower risk of pre-delivery hospitalisation with influenza like illness compared to unvaccinated women (aHR 0•61; 95% CI 0•39, 0•97) (Table 2). After accounting for the assumption that immunologic protection after influenza vaccination requires 2 weeks for full effect, the estimated aHR remained unchanged (supplementary material p 2). The observed protective effect of maternal influenza vaccination in reducing hospitalisation due to influenza like illness was stronger for those vaccinated in second trimester (aHR 0•09; 95% CI 0•01, 0•71) and those who delivered during periods of high influenza activity (aHR 0•51; 95% CI 0•27, 0•95) (Table 3).

**Table 2:** Crude and adjusted hazard ratios for time-based pregnancy and birth outcomes by maternal influenza vaccination status at two obstetric hospitals in South Australia 2015–2018.

Variables	Total	Unvaccinated N (%)	Vaccinated N (%)	Crude HR* (95% CI)	p-value	Adjusted † aHR (95% CI)	p-value
Pre-delivery hospitalisation due to influenza like illness ‡	95/1253 (7•5)	60/650 (9•2)	35/603 (5•8)	0•58 (0•37, 0•91)	0•018	0•61 (0•39, 0•97)	0•038
Spontaneous abortion §	34/1253 (2•7)	31/650 (4•7)	3/603 (0•5)	0•66 (0•20, 2•19)	0•507	0•42 (0•12, 1•45)	0•171
Gestational hypertension ||	81/1205 (6•7)	41/606 (6•7)	40/599 (6•6)	0•80 (0•49, 1•31)	0•391	0•78 (0•47, 1•29)	0•343
Pre-eclampsia ||	111/1205 (9•2)	58/606 (9•5)	53/599 (8•8)	0•85 (0•58, 1•26)	0•445	0•84 (0•54, 1•27)	0•417
Severe pre-eclampsia ||	28/1204 (2•3)	14/606 (2•3)	14 /598 (2•3)	0•86 (0•37, 1•96)	0•725	0•65 (0•26, 1•64)	0•368
Gestational diabetes ¶	190/1207 (15•7)	85/608 (13•9)	105/599 (17•5)	1•33 (0•95, 1•84)	0•088	1•16 (0•82, 1•66)	0•383
Preterm premature rupture of the membranes ⁎⁎	47/1207 (3•8)	27/608 (4•4)	20/599 (3•3)	0•82 (0•43, 1•56)	0•561	0•85 (0•44, 1•63)	0•634
Preterm birth ⁎⁎	89/1207 (7•3)	49/608 (8•0)	40/599 (6•6)	0•94 (0•60, 1•47)	0•802	0•94 (0•59, 1•49)	0•817
Spontaneous preterm birth ⁎⁎	59/1207 (4•8)	36/608(5•9)	23/599 (3•8)	0•71 (0•40, 1•26)	0•253	0•74 (0•41, 1•33)	0•323
LBW (<2500 g) ††	80/1205 (6•6)	49/606 (8•0)	31/599 (5•1)	0•70 (0•42, 1•14)	0•158	0•71 (0•43, 1•19)	0•202
LBW at term (<2500 g)††^,^‡‡	29/1116 (2•6)	20/557 (3•5)	9 /559 (1•6)	0•43 (0•18, 0•99)	0•048	0•38 (0•16, 0•89)	0•027
SGA ††	144/1207 (11•9)	83 /608(13•6)	61/599 (10•1)	0•77 (0•54, 1•09)	0•152	0•84 (0•58, 1•20)	0•346
				**Difference in means (vaccinated- unvaccinated)**		**Difference in adjusted means (vaccinated -unvaccinated)**	
Mean birth weight ††, g (95% CI)	3334•9 ± 557	3301•8 ± 610	3368•4 ± 498	63•7 (0•09, 127•0)	0•050	58•8 (- 4•2, 121•7)	0•067
Mean gestational age at delivery, weeks (95% CI)	39•2 ± 2•0	39•1 ± 2•3	39•4 ± 1•6	0•26 (0•04, 0•49)	0•019	0•27 (0•04, 0•49)	0•019

CI=confidence interval. HR=hazard ratios. LBW=low birthweight. SGA=small for gestational age.

⁎ HR results compared outcome variable in vaccinated group to reference (unvaccinated).

† Adjustments were made for maternal age, race/ethnicity, education, household income, gravidity, intake of alcohol and recreational drugs, smoking, pre-pregnancy body mass index (continuous), use of multivitamin supplements, Edinburgh Postnatal. Depression Scale (EPDS), The State-Trait Anxiety Inventory (STAI), Perceived Stress Scale (PSS-10), physical activity, infertility treatment, asthma and estimated season of delivery.

‡ Women admitted to hospital with influenza/ respiratory tract infection were censored at their admission date.

§ The time metric for spontaneous abortion analysis was the first week of gestation up to the event (week of last available pregnancy data or week 20 of gestation; whichever occurred first).

|| For hypertensive disorders analysis, women who were vaccinated at or after the gestational age at diagnosis (≥ 20 weeks' gestation) and pregnancies ending prior to 20 weeks of gestation were censored.

¶ Women who were vaccinated at or after the gestational age at diagnosis of gestational diabetes mellitus (median gestational age at screening was 27•8 (IQR, 26•5–29) weeks) were censored.

⁎⁎ Women vaccinated at 37 weeks’ or later were censored because they were no longer at risk of preterm birth.

†† Additionally adjusted for infant's sex.

‡‡ Low birthweight at term (<2500 g and ≥ 37 completed weeks’ gestation at birth).

**Table 3:** Crude and adjusted hazard ratios for pre-delivery hospitalisation due to influenza like illness and key adverse birth outcomes stratified by trimester of influenza vaccination and influenza activity.

Variables	Unvaccinated N (%)	Vaccinated N (%)	Crude HR* (95% CI)	p-value	Adjusted† aHR (95% CI)	p-value
Pre-delivery hospitalisation due to influenza like illness 1st trimester 2nd trimester 3rd trimester Low influenza activity High influenza activity	60/650 (9•2)	35/603 (5•8)	0•58 (0•37, 0•91) 0•43 (0•18, 0•99) 0•09 (0•01, 0•68) 0•70 (0•43, 1•13) 0•58 (0•33, 0•99) 0•47 (0•26, 0•85)	0•018 0•049 0•019 0•149 0•049 0•013	0•61 (0•39, 0•97) 0•46 (0•19, 1•09) 0•09 (0•01, 0•72) 0•73 (0•44, 1•22) 0•60 (0•35, 1•05) 0•53 (0•29, 0•96)	0•038 0•080 0•023 0•244 0•079 0•039
Preterm birth‡ 1st trimester 2nd trimester 3rd trimester Low influenza activity High influenza activity	49/608 (8•0)	40/599 (6•6)	0•94 (0•60, 1•47) 0•46 (0•18, 1•16) 0•91 (0•43, 1•93) 0•79 (0•47, 1•33) 0•61 (0•33, 1•12) 0•85 (0•50, 1•45)	0•802 0•111 0•811 0•384 0•112 0•571	0•94 (0•59, 1•49) 0•50 (0•19, 1•28) 0•90 (0•41, 1•94) 0•75 (0•43, 1•29) 0•61 (0•33, 1•12) 0•89 (0•52, 1•53)	0•817 0•151 0•793 0•304 0•113 0•696
Spontaneous preterm birth ‡ 1st trimester 2nd trimester 3rd trimester Low influenza activity High influenza activity	36/608(5•9)	23/599 (3•8)	0•71 (0•40, 1•26) 0•37 (0•11, 1•22) 0•61 (0•21, 1•73) 0•61 (0•31, 1•18) 0•53 (0•25, 1•11) 0•58 (0•28, 1•17)	0•253 0•104 0•361 0•147 0•096 0•131	0•74 (0•41, 1•33) 0•41 (0•12, 1•37) 0•61 (0•21, 1•76) 0•63 (0•32, 1•25) 0•55 (0•26, 1•17) 0•60 (0•29, 1•26)	0•323 0•149 0•364 0•193 0•123 0•180
LBW (<2500 g)§ 1st trimester 2nd trimester 3rd trimester Low influenza activity High influenza activity	49/606 (8•0)	31/599 (5•1)	0•70 (0•42, 1•14) 0•56 (0•24, 1•35) 0•47 (0•16, 1•30) 0•57 (0•32, 1•03) 0•66 (0•37, 1•19) 0•43 (0•22, 0•86)	0•158 0•206 0•161 0•065 0•171 0•018	0•71 (0•43, 1•19) 0•54 (0•23, 1•29) 0•41 (0•14, 1•16) 0•63 (0•34, 1•15) 0•63 (0•34, 1•14) 0•46 (0•23, 0•94)	0•202 0•168 0•096 0•138 0•170 0•033
LBW at term (<2500 g)§^,^||^,^¶ Low influenza activity High influenza activity	20/557 (3•5)	9 /559 (1•6)	0•43 (0•18,1•09) 0•61 (0•24, 1•54) 0•20 (0•05, 0•89)	0•048 0•303 0•035	0•38 (0•16, 0•89) 0•48 (0•18, 1•36) 0•20 (0•04, 0•87)	0•027 0•132 0•032
SGA§ 1st trimester 2nd trimester 3rd trimester Low influenza activity High influenza activity	83 /608 (13•6)	61/599 (10•1)	0•77 (0•55, 1•09) 1•15 (0•71, 1•86) 0•79 (0•42, 1•49) 0•57 (0•36, 0•89) 0•88 (0•59, 1•32) 0•60 (0•37, 0•95)	0•152 0•549 0•476 0•014 0•596 0•030	0•84 (0•58, 1•20) 1•22 (0•74, 2•02) 0•79 (0•41, 1•50) 0•61 (0•38, 0•98) 0•92 (0•61, 1•39) 0•65 (0•40, 1•04)	0•346 0•347 0•483 0•044 0•708 0•079

CI=confidence interval. HR=hazard ratios. LBW=low birthweight. SGA=small for gestational age.

⁎ HR results compared outcome variable in vaccinated group to reference (unvaccinated).

† Adjustments were made for maternal age, race/ethnicity, education, household income, gravidity, intake of alcohol and recreational drugs, smoking, pre-pregnancy body mass index (continuous), use of multivitamin supplements, Edinburgh Postnatal Depression Scale (EPDS), The State-Trait Anxiety Inventory (STAI), Perceived Stress Scale (PSS-10), physical activity, infertility treatment, asthma and estimated season of delivery.

‡ Women vaccinated at 37 weeks’ or later were censored because they were no longer at risk of having a preterm birth.

§ Additionally adjusted for infant's sex.

|| Low birthweight at term (<2500 g and ≥ 37 completed weeks’ gestation at birth).

¶ Analysis by trimester of influenza vaccination was not performed because a small number of mothers who delivered LBW at term babies received the vaccine prior to their third trimester (*n* = 1 during 1st trimester, *n* = 1 during 2nd trimester).

There was no association with spontaneous abortion for women who were vaccinated for influenza prior to 20 weeks’ gestation (aHR 0•42, 95% CI 0•12, 1•45) (Table 2). Our Cox model shows that influenza vaccination during pregnancy was not associated with maternal hypertensive disorders including gestational hypertension (aHR 0•78, 95% CI 0•47, 1•29), pre-eclampsia (aHR 0•84, 95% CI 0•54, 1•27) or severe pre-eclampsia (aHR 0•65, 95% CI 0•26, 1•64) (Table 2). Additional adjustment for maternal pertussis vaccination as a time-varying covariate yielded similar results for hypertensive disorders (supplementary material p 2). In the log-binomial models, there was no association between risk of chorioamnionitis and influenza vaccination during pregnancy (aRR 0•78, 95% CI, 0•32, 1•88) (Table 4).

**Table 4:** Pregnancy and birth outcomes following influenza vaccination in pregnancy at two obstetric hospitals in South Australia 2015–2018.

Pregnancy outcomes	Total	Unvaccinated N (%)	Vaccinated N (%)	Risk Ratios RR (95% CI)	p-value	Adjusted* aRR (95% CI)	p-value
Chorioamnionitis and/or funisitis	25/1207 (2•0)	15/608 (2•4)	10/599 (1•6)	0•65 (0•28, 1•49)	0•316	0•78 (0•32, 1•88)	0•581
Postpartum haemorrhage	113/1205 (9•3)	62/606 (10•2)	51/599 (8•5)	0•79 (0•55, 1•14)	0•215	0•72 (0•49, 1•06)	0•099
Caesarean delivery (Vs Vaginal)†	349/1205 (28•9)	176/606 (29•0)	173/599 (28•8)	1•01 (0•93, 1•08)	0•758	0•91 (0•75, 1•09)	0•326
**Birth outcomes**							
Congenital anomalies‡	23/1207 (1•9)	21/1066 (1•8)	2/141 (1•4)	0•31 (0•04, 2•33)	0•256	0•33 (0•04, 2•73)	0•311
Low Apgar at 1 min (<7)	151/1201 (12•5)	72/603 (11•9)	79/598 (13•2)	1•13 (0•83, 1•53)	0•433	1•11 (0•81, 1•52)	0•490
Low Apgar at 5-min (<7)	31/1203 (2•5)	16/604 (2•6)	15/599 (2•5)	0•93 (0•44, 1•97)	0•874	0•84 (0•39, 1•81)	0•670
Admitted to Neonatal unit§	282/1207 (23•3)	140/608 (23•0)	142/599 (23•7)	0•98 (0•80, 1•22)	0•780	1•04 (0•84, 1•28)	0•693
Respiratory distress syndrome	14/1207 (1•1)	10/608 (1•6)	4/599 (0•6)	0•40 (0•12, 1•26)	0•120	0•46 (0•14, 1•52)	0•208
Mechanical ventilation	51/1207 (4•2)	30/608 (4•9)	21/599 (3•5)	0•72 (0•41, 1•26)	0•258	0•74 (0•42, 1•31)	0•313

⁎ Pregnancy outcomes were adjusted for maternal age, ethnicity, total years of full time education, household income, gravidity, intake of alcohol and recreational drugs, smoking, pre-pregnancy body mass index (continuous), use of multivitamin supplements, Edinburgh Postnatal Depression Scale (EPDS), The State-Trait Anxiety Inventory (STAI), Perceived Stress Scale (PSS-10), physical activity, infertility treatment, asthma and estimated season of delivery• Birth outcomes were additionally adjusted for infant's sex.

† Poisson regression model was used because the log binomial model failed to converge.

‡ For congenital anomalies analysis, the exposure time window comprised the first trimester and women vaccinated after first trimester were classified as unvaccinated.

§ Reasons for admission: Preterm, Respiratory distress Infection, Feeding problem, Hypoglycaemia, Drug withdrawal, SGA, Birth asphyxia, congenital abnormality, Phototherapy and Cyanosis.

After adjusting for covariates, women vaccinated for influenza during pregnancy had on average 1•8 days longer gestation at delivery than unvaccinated women (Table 2). Restricting the analysis to pregnancies reaching at least 32 weeks’ gestation followed by adjustment for maternal pertussis vaccination showed that any differences in gestational age at birth between unvaccinated and vaccinated mothers were negligible (supplementary material p 2). Overall, 7•3% (89 of 1207) of pregnancies resulted in preterm birth. There was no difference in stillbirth between vaccinated (*n* = 3) and unvaccinated women (*n* = 3). Our time-dependant analysis showed no association between influenza vaccination through to 37 weeks’ gestation and preterm birth (aHR 0•94, 95% CI 0•59, 1•49), preterm premature rupture of the membranes (aHR 0•85, 95% CI 0•44, 1•63), and spontaneous preterm birth (aHR 0•74, 95% CI 0•41, 1•33) (Table 2). Maternal influenza vaccination showed a modest reduction in the hazard of spontaneous preterm birth during periods of lower influenza virus circulation but the confidence intervals were wide and included one (aHR 0•52, 95% CI 0•24, 1•13) (supplementary material p 3).

### 3.2 Birth outcomes

Maternal influenza vaccination was protective against delivering LBW term infants in our Cox proportional hazard regression analyses (aHR 0•38, 95% CI 0•16, 0•89) (Table 2). This effect persisted following additional adjustment for maternal pertussis vaccination (aHR 0•38, 95% CI 0•15, 0•94) (supplementary material p 2). An even greater protective effect of influenza vaccination against delivering a LBW infant at term (aHR 0•20, 95% CI 0•04, 0•87) and LBW in either preterm or term infants (aHR 0•46, 95% CI 0•23, 0•94) was observed during periods of high influenza activity (Table 3). There was no evidence of increased risk of LBW associated with receipt of inactivated influenza vaccine during any trimester of pregnancy (Table 3). First trimester influenza vaccination had no effect on risk of congenital anomalies (aRR 0•33, 95% CI 0•04, 2•73) (Table 4). Overall, 510 (42•2%) of 1207 infants were born during high influenza activity across three Australian influenza seasons 2015–2018. The infants born to vaccinated mothers were estimated to be 59 g heavier than infants born to unvaccinated mothers (58•8 g, 95% CI −4•2 g, 121•7 g) but the confidence intervals were wide and included zero (Table 2). This association was attenuated (18•3 g, 95% CI – 42•2 g, 79•0 g) after adjustment for maternal pertussis vaccination (supplementary material p 2).

Our study found no increased risk for SGA delivery after influenza vaccination during pregnancy (aHR 0•84, 95% CI 0•58, 1•20) (Table 2). Maternal influenza vaccination was associated with a marginal reduction in risk of SGA births during periods of high influenza activity (aHR 0•65, 95% CI 0•40, 1•04). Influenza vaccination in third trimester was associated with a 39% reduction in risk of SGA birth regardless of the level of influenza activity (aHR 0•61, 95% CI 0•38, 0•98) (Table 3). However, these protective effects on SGA were slightly attenuated after adjustment for pertussis vaccination (supplementary material p 3). There was no association between maternal influenza vaccination and adverse infant outcomes including low Apgar scores at 1 and 5 min, admission to the neonatal care unit, mechanical ventilation, and respiratory distress syndrome (Table 4).

## 4 Discussion

In robust nuanced analyses that account for timing of maternal influenza vaccination and the time risk of adverse pregnancy outcomes, we show maternal influenza vaccination is safe in a prospective cohort of healthy pregnant women, with confirmed vaccination history and accurate, pregnancy and infant outcome data. There was no evidence of associations between influenza vaccination administered at any time in pregnancy and adverse pregnancy or foetal outcomes including spontaneous abortion, congenital anomalies, shortened gestation, gestational diabetes, chorioamnionitis or gestational hypertensive disorders, consistent with the literature [8], [9], [10], [11], [12]. In addition to reassuring safety of maternal influenza vaccination, our study found influenza vaccination during pregnancy reduced a pregnant woman's risk of pre-delivery hospitalisation with influenza like illness by around 39%. This protective effect was most pronounced for those women who delivered during periods of high influenza activity, consistent with previous studies [20, 21]. Across the three influenza seasons 2015–2018 in South Australia, influenza A (H3N2) was the dominant circulating virus followed by influenza B [17].

In contrast to our findings, a recent Bayesian meta-analysis of 28 cohort studies showed maternal influenza vaccination protects against preterm birth [13]. However, the pooled summary estimates [13] did not find any association when the preterm birth analysis included 2 randomized placebo-controlled studies (RCTs) and 2 case-control studies. The two RCTs [22, 23] investigating maternal influenza vaccine efficacy and safety in South Africa and Nepal, respectively, found that vaccination was not associated with preterm birth. However, the RCT in Nepal showed a reduction of LBW [23] and another RCT [7] conducted in Bangladesh demonstrated a reduction of SGA amongst a subset of infants born during peak influenza circulation to influenza-vaccinated women. We also found that vaccinated mothers were less likely to deliver LBW and SGA infants during periods of high influenza activity. Decreased risk for LBW and SGA during peak influenza season amongst vaccinated mothers could be attributed to decreased risk of influenza infection during pregnancy following maternal influenza vaccination. Differing from our study findings, a secondary analysis of the RCT in Nepal [24], which was the only trial powered to detect difference in birth weight has found that maternal influenza vaccination significantly increased mean birthweight by 42 g. Birth weight is an important indicator of an infant's vulnerability to the risk of childhood illness and chances of survival and the health burden of babies born SGA or LBW is very high in low income countries [25]. Reduction of these adverse birth outcomes following maternal influenza vaccination would be an important achievement, particularly in tropical regions, where influenza circulates year-round.

Consistent with previous studies, [26, 27] we demonstrated that newborns whose mothers were vaccinated for influenza in pregnancy were not more likely to experience any adverse outcomes, including admission to the neonatal care unit, respiratory distress, low Apgar scores nor need for mechanical ventilation at birth compared with neonates born to unvaccinated women. A protective effect of maternal influenza vaccination on preventing either influenza or influenza-related complications in infants up to 6 months old [28, 29] provides important additional evidence that women should be offered influenza vaccination during pregnancy, irrespective of time of year.

Our study has a number of strengths and some potential limitations. The major strength is the prospective cohort design that recruited a large number of nulliparous women with singleton pregnancies at low risk for obstetric complications at two major maternity hospitals, reducing potential confounding by indication. Such bias could have occurred if women with known comorbidities and/or high-risk factors were more likely to receive the influenza vaccine during pregnancy and have a higher baseline risk of adverse pregnancy outcomes than healthy women leading to an underestimation of vaccine safety. The opposite effect (i.e. an overestimate of the size of the protective effect of maternal vaccination) due to a ‘healthy vaccinee bias’ could also have occurred. Vaccinated women in our study were more likely to engage in healthy lifestyles i.e. pregnancy micronutrient supplementation, exercise regularly and were less likely to smoke or use illicit drugs in pregnancy than unvaccinated women. The analysis framework used herein adjusted for putative risk factors, including psychosocial factors, to mitigate the impact of any ‘healthy vaccinee bias’ on our findings.

Our use of Cox proportional-hazards models accounting for time-varying vaccine exposure within pregnancy, minimized the introduction of immortal time bias in our data [30]. The potential for this bias arises because the opportunity for vaccination increases the longer a woman remains pregnant and free of adverse foetal outcomes. The fact that the pregnancies were not followed from the beginning (i.e. first day of the last menstrual period), causes downward bias in estimation of spontaneous abortion. Such data are said to be left truncated. Additionally, including follow-up time during which pregnancies are no longer at risk of some adverse outcomes (e.g. gestation after 37 weeks’ considered for preterm birth outcomes) can lead to overestimation of any true benefits of maternal vaccination but our analysis strategy minimized the risk of these biases occurring. One potential limitation that we could not take into account is that vaccine administered in non-traditional settings (i.e. pharmacist or community or workplace-administered vaccination) might not be recorded in women's Pregnancy-Hand-Held-Records. Thus, uptake of vaccination during pregnancy may have been underestimated. However, this is unlikely as women were interviewed by a research midwife at several time points including post-delivery to confirm final vaccination status. Another limitation in our study is the inability to distinguish pre-delivery hospital admission due to laboratory-confirmed influenza infections from influenza-like illness. However, these limitations are likely to have negligible effects on our study findings.

Evidence from previous influenza pandemics, and seasonal influenza demonstrates that pregnant women and their infants are at high risk of severe influenza-related complications [1, 2]. Our robust study analysis demonstrated that maternal influenza vaccination reduced pregnant women's risk of pre-delivery hospitalisation with influenza like illness. Furthermore, our study provides a unique prospective assessment of the safety of an inactivated influenza vaccine amongst pregnant women providing reassurance for health providers and pregnant women. Importantly, although numerous factors may contribute, we show positive impacts on key birth outcomes that inordinately occur in low-middle income countries with long term consequences for offspring health and impacts on low capacity health systems.

## Data statement

The datasets generated and/or analysed during the current study are available upon reasonable request to Prof. Claire Roberts (claire.roberts@adelaide.edu.au) and subject to regulatory approvals.

## Declaration of Competing Interest

HSM has been an investigator on clinical trials funded by pharmaceutical companies including Pfizer, GSK Sanofi-Pasteur, Novartis. Her institution receives funding for Investigator led research. HSM receives no personal payments from Industry. The remaining authors have no conflicts of interest to declare.

## Appendix Supplementary materials

Image, application 1

Image, application 2
