# Supplementary material for: Safety and protective effects of maternal influenza vaccination on pregnancy and birth outcomes: A prospective cohort study
Source: eClinicalMedicine. 2020 Sep 9;26:100522. doi: 10.1016/j.eclinm.2020.100522 (PMC7490992; doi:10.1016/j.eclinm.2020.100522)
Supplement: Supplementary file 1 [file mmc1.docx]

**Title:** **Safety and protective effects of maternal influenza vaccination on pregnancy and birth outcomes****: A prospective cohort study**

**Authors:** Hassen Mohammed, BHSc-Hons,^1,2,3^ Claire T Roberts, PhD ^2,3,4^, Luke E Grzeskowiak, PhD,^2,3,5^ Lynne Giles, PhD,^2,3,6^ Gustaaf A Dekker, MD,^2,3,7^ Helen S Marshall, MD,^1,2,3,6^

**Supplementary Material:**

**Supplementary Table 1:** **Comparison of women included and excluded from the study (for whom data are available) in terms of key variables of interest**

| Key Variables | Excluded  n/N (%) | Included  n/N (%) |
| --- | --- | --- |
| Pre-delivery hospitalisation due to influenza like illness | 5/93 (5.3) | 95/1253 (7.5) |
| Spontaneous abortion | 5/93 (5.3) | 34/1253 (2.7) |
| Gestational hypertension | 5/76 (6.5) | 81/1205 (6.7) |
| Pre-eclampsia | 10/77 (12.9) | 111 /1205 (9.2) |
| Preterm birth | 7/79 (8.8) | 89/1207 (7.3) |
| LBW | 5/77 (6.4) | 80/1205 (6.6) |
| LBW at term | 2/70 (2.8) | 29 /1116 (2.6) |
| SGA | 11/76 (14.4) | 142/1203 (11.8) |

LBW=low birthweight. SGA=small for gestational age.

**Supplementary Table 2:** Time-based pregnancy and birth outcomes by maternal influenza vaccination status after controlling for the receipt of a pertussis vaccine in pregnancy at two obstetric hospitals in South Australia 2015-2018

| Variables | Total | Unvaccinated  N (%) | Vaccinated  N (%) | Crude  HR ^*^ (95% CI) | p-value | Adjusted ^†^  aHR (95% CI) | p-value |
| --- | --- | --- | --- | --- | --- | --- | --- |
| Pre-delivery hospitalisation due to influenza like illness ^‡^  Accounting the 2-weeks period required for full protection | 95/1253 (7·5) | 60/650 (9·2) | 35/603 (5·8) | 0·58 (0·37, 0·91)  0·58 (0·37, 0·91) | 0·018  0·021 | 0·60 (0·37, 0·97)  0·60 (0·37, 0·98) | 0·040  0·044 |
| Gestational hypertension ^§^ | 81/1205 (6·7) | 41/606 (6·7) | 40/599 (6·6) | 0·80 (0·49, 1·31) | 0·391 | 0·72 (0·43, 1·21) | 0·299 |
| Pre-eclampsia ^§^ | 111/1205 (9·2) | 58/606 (9·5) | 53/599 (8·8) | 0·85 (0·58, 1·26) | 0·445 | 0·89 (0·57, 1·38) | 0·622 |
| Severe pre-eclampsia ^§^ | 28/1204 (2·3) | 14/606 (2·3) | 14 /598 (2·3) | 0·86 (0·37, 1·96) | 0·725 | 0·68 (0·25, 1·81) | 0·450 |
| Gestational diabetes ^\|\|^ | 190/1207 (15·7) | 85/608 (13·9) | 105/599 (17·5) | 1·33 (0·95, 1·84) | 0·088 | 1·10 (0·75, 1·60) | 0·611 |
| Preterm premature rupture of the membranes ^¶^ | 47/1207 (3·8) | 27/608 (4·4) | 20/599 (3·3) | 0·82 (0·43, 1·56) | 0·561 | 0·90 (0·44, 1·82) | 0·722 |
| Preterm birth ^¶^ | 89/1207 (7·3) | 49/608 (8·0) | 40/599 (6·6) | 0·94 (0·60, 1·47) | 0·802 | 1·01 (0·61, 1·66) | 0·951 |
| Spontaneous preterm birth ^¶^ | 59/1207 (4·8) | 36/608(5·9) | 23/599 (3·8) | 0·71 (0·40, 1·26) | 0·253 | 0·71 (0·38, 1·32) | 0·287 |
| LBW (<2500 g) ^**^ | 80/1205 (6·6) | 49/606 (8·0) | 31/599 (5·1) | 0·70 (0·42, 1·14) | 0·158 | 0·81 (0·47, 1·39) | 0·450 |
| LBW at term(<2500 g) ^**, ††^ | 29/1116 (2·6) | 20/557 (3·5) | 9 /559 (1·6) | 0·43 (0·18, 0·99) | 0·048 | 0·38 (0·15, 0·94) | 0·037 |
| SGA ** | 144/1207 (11·9) | 83 /608(13·6) | 61/599 (10·1) | 0·77 (0·54, 1·09) | 0·152 | 0·91 (0·62, 1·34) | 0·710 |
|  |  |  |  | **Difference in means (vaccinated-unvaccinated)** | | **Difference in adjusted means (vaccinated - unvaccinated)** | |
| Mean birth weight ^h^, g  (95% CI) | 3360·3± 505 | 3343·7 ± 526 | 3376·9 ± 484 | 33·1 (-24·3, 90·7) | 0·258 | 18·3 (- 42·2, 79·0) | 0·552 |
| Mean gestational age at delivery, weeks (95% CI)) | 39·4 ± 1·5 | 39·3 ± 1·5 | 39·4 ± 1·5 | 0·07 (-0·09, 0·25) | 0·381 | 0·05 (-0·13, 0·24) | 0·570 |

CI=confidence interval. HR=hazard ratios. LBW=low birthweight. SGA=small for gestational age.

* HR results compared outcome variable in vaccinated group to reference (unvaccinated).

† Adjustments were made for pertussis vaccine in pregnancy, maternal age, race/ethnicity, education, household income, gravidity, intake of alcohol and recreational drugs, smoking, pre-pregnancy body mass index (continuous), use of multivitamin supplements, Edinburgh Postnatal· Depression Scale (EPDS), The State-Trait Anxiety Inventory (STAI), Perceived Stress Scale (PSS-10), physical activity, infertility treatment, asthma and estimated season of delivery.

‡ Women admitted to hospital with influenza/ acute respiratory tract infection were censored at their admission date.

§ For hypertensive disorders analysis, women who were vaccinated at or after the gestational age at diagnosis (≥ 20 weeks' gestation) and pregnancies ending prior to 20 weeks of gestation were censored.

|| Women who were vaccinated at or after the gestational age at diagnosis of gestational diabetes mellitus (median gestational age at screening was 27·8 (IQR, 26·5-29) weeks) were censored.

¶ Women vaccinated at 37 weeks’ or later were censored because they were no longer at risk of preterm birth.

** Additionally adjusted for infant's sex.

†† Low birthweight at term (<2500 g and ≥ 37 completed weeks’ gestation at birth).

**Supplementary Table 3:** Crude and adjusted hazard ratios for pre-delivery hospitalisation due to influenza like illness and key adverse birth outcomes stratified by trimester of influenza vaccination and influenza activity after controlling for the receipt of maternal pertussis vaccination.

| Variables | Unvaccinated  N (%) | Vaccinated  N (%) | Crude  HR * (95% CI) | p-value | Adjusted ^†,^ ^‡^  aHR (95% CI) | p-value |
| --- | --- | --- | --- | --- | --- | --- |
| Pre-delivery hospitalisation due to influenza like illness  1^st^ trimester  2^nd^ trimester  3^rd^ trimester  Low influenza activity  High influenza activity | 60/650 (9·2) | 35/603 (5·8) | 0·58 (0·37, 0·91)  0·43 (0·18, 0·99)  0·09 (0·01, 0·68)  0·70 (0·43, 1·13)  0·58 (0·33, 0·99)  0·47 (0·26, 0·85) | 0·018  0·049  0·019  0·149  0·049  0·013 | 0·60 (0·37, 0·97)  0·45 (0·19, 1·08)  0·09 (0·01, 0·71)  0·71 (0·41, 1·22)  0·59 (0·33, 1·04)  0·51 (0·27, 0·95) | 0·040  0·075  0·022  0·224  0·070  0·035 |
| Preterm birth ^§^  1^st^ trimester  2^nd^ trimester  3^rd^ trimester  Low influenza activity  High influenza activity | 49/608 (8·0) | 40/599 (6·6) | 0·94 (0·60, 1·47)  0·46 (0·18, 1·16)  0·91 (0·43, 1·93)  0·79 (0·47, 1·33)  0·61 (0·33, 1·12)  0·85 (0·50, 1·45) | 0·802  0·111  0·811  0·384  0·112  0·571 | 1·01 (0·61 1·66)  0·51 (0·19, 1·32)  0·93 (0·42, 2·03)  0·77 (0·44, 1·37)  0·62 (0·34, 1·17)  0·93 (0·53, 1·63) | 0·951  0·167  0·859  0·390  0·143  0·807 |
| Spontaneous preterm birth ^§^  1^st^ trimester  2^nd^ trimester  3^rd^ trimester  Low influenza activity  High influenza activity | 36/608(5·9) | 23/599 (3·8) | 0·71 (0·40, 1·26)  0·37 (0·11, 1·22)  0·61 (0·21, 1·73)  0·61 (0·31, 1·18)  0·53 (0·25, 1·11)  0·58 (0·28, 1·17) | 0·253  0·104  0·361  0·147  0·096  0·131 | 0·71 (0·38, 1·32)  0·39 (0·11, 1·32)  0·57 (0·20, 1·69)  0·59 (0·29, 1·21)  0·52 (0·24, 1·13)  0·56 (0·26, 1·21) | 0·287  0·134  0·318  0·156  0·101  0·143 |
| LBW (<2500 g) ^\|\|^  1^st^ trimester  2^nd^ trimester  3^rd^ trimester  Low influenza activity  High influenza activity | 49/606 (8·0) | 31/599 (5·1) | 0·70 (0·42, 1·14)  0·56 (0·24, 1·35)  0·47 (0·16, 1·30)  0·57 (0·32, 1·03)  0·66 (0·37, 1·19)  0·43 (0·22, 0·86) | 0·158  0·206  0·161  0·065  0·171  0·018 | 0·81 (0·47, 1·39)  0·58 (0·24, 1·41)  0·44 (0·15, 1·28)  0·66 (0·35, 1·22)  0·67 (0·36, 1·24)  0·51 (0·24, 1·05) | 0·450  0·233  0·137  0·191  0·255  0·068 |
| LBW at term  (<2500 g) ^\|\|, ¶ , **^  Low influenza activity  High influenza activity | 20/557 (3·5) | 9 /559 (1·6) | 0·43 (0·18,1·09)  0·61 (0·24, 1·54)  0·20 (0·05, 0·89) | 0·048  0·303  0·035 | 0·38 (0·15, 0·94)  0·47 (0·17, 1·26)  0·19 (0·04, 0·88) | 0·037  0·139  0·034 |
| SGA ^e^  1^st^ trimester  2^nd^ trimester  3^rd^ trimester  Low influenza activity  High influenza activity | 83 /608 (13·6) | 61/599 (10·1) | 0·77 (0·55, 1·09)  1·15 (0·71, 1·86)  0·79 (0·42, 1·49)  0·57 (0·36, 0·89)  0·88 (0·59, 1·32)  0·60 (0·37, 0·95) | 0·152  0·549  0·476  0·014  0·596  0·030 | 0·91 (0·62, 1·34)  1·27 (0·77, 2·11)  0·84 (0·43, 1·61)  0·66 (0·40, 1·08)  0·98 (0·64, 1·50)  0·71 (0·43, 1·16) | 0·710  0·335  0·603  0·105  0·941  0·175 |

CI=confidence interval. HR=hazard ratios. LBW=low birthweight. SGA=small for gestational age.

* HR results compared outcome variable in vaccinated group to reference (unvaccinated).

† Adjustments were made for maternal age, race/ethnicity, education, household income, gravidity, intake of alcohol and recreational drugs, smoking, pre-pregnancy body mass index (continuous), use of multivitamin supplements, Edinburgh Postnatal· Depression Scale (EPDS), The State-Trait Anxiety Inventory (STAI), Perceived Stress Scale (PSS-10), physical activity, infertility treatment, asthma and estimated season of delivery.

‡ Additionally adjusted for receipt of pertussis vaccine during pregnancy.

§ Women vaccinated at 37 weeks’ or later were censored because they were no longer at risk of having a preterm birth.

|| Additionally adjusted for infant's sex.

¶ Low birthweight at term (<2500 g and ≥ 37 completed weeks’ gestation at birth).

** Analysis by trimester of influenza vaccination was not performed because a small number of mothers who delivered LBW at term babies received the vaccine prior to their third trimester (n=1 during 1st trimester, n =1 during 2nd trimester).

**Supplementary Table 4:** Pregnancy and birth outcomes following influenza vaccination in pregnancy after controlling for the receipt of a pertussis vaccine in pregnancy at two obstetric hospitals in South Australia 2015-2018

| Pregnancy outcomes | Total | Unvaccinated  N (%) | Vaccinated  N (%) | Risk Ratios  RR (95% CI) | p-value | Adjusted ^*, †^  aRR (95% CI) | p-value |
| --- | --- | --- | --- | --- | --- | --- | --- |
| Chorioamnionitis and/or funisitis | 25/1207 (2·0) | 15/608 (2·4) | 10/599 (1·6) | 0·65 (0·28, 1·49) | 0·316 | 0·93 (0·36, 2·43) | 0·893 |
| Postpartum haemorrhage | 113/1205 (9·3) | 62/606 (10·2) | 51/599 (8·5) | 0·79 (0·55, 1·14) | 0·215 | 0·73 (0·49, 1·09) | 0·129 |
| Caesarean delivery (Vs Vaginal) ^‡^ | 349/1205 (28·9) | 176/606 (29·0) | 173/599 (28·8) | 1·01 (0·93, 1·08) | 0·758 | 0·91 (0·79, 1·05) | 0·239 |
| Birth outcomes |  |  |  |  |  |  |  |
| Low Apgar at 1 min (<7) | 151/1201 (12·5) | 72/603 (11·9) | 79/598 (13·2) | 1·13 (0·83, 1·53) | 0·433 | 1·26 (0·90, 1·77) | 0·160 |
| Low Apgar at 5-min (<7) | 31/1203 (2·5) | 16/604 (2·6) | 15/599 (2·5) | 0·93 (0·44, 1·97) | 0·874 | 1·05 (0·46, 2·39) | 0·902 |
| Admitted to Neonatal unit ^§^ | 282/1207 (23·3) | 140/608 (23·0) | 142/599 (23·7) | 0·98 (0·80, 1·22) | 0·780 | 1·12 (0·89, 1·40) | 0·309 |
| Respiratory distress syndrome | 14/1207 (1·1) | 10/608 (1·6) | 4/599 (0·6) | 0·40 (0·12, 1·26) | 0·120 | 0·78 (0·20, 2·95) | 0·722 |
| Mechanical ventilation | 51/1207 (4·2) | 30/608 (4·9) | 21/599 (3·5) | 0·72 (0·41, 1·26) | 0·258 | 0·97 (0·52, 1·81) | 0·933 |

* Pregnancy outcomes were adjusted for maternal age, ethnicity, total years of full time education, household income, gravidity, intake of alcohol and recreational drugs, smoking, pre-pregnancy body mass index (continuous) , use of multivitamin supplements, Edinburgh Postnatal· Depression Scale (EPDS), The State-Trait Anxiety Inventory (STAI), Perceived Stress Scale (PSS-10), physical activity, infertility treatment, asthma and estimated season of delivery. Birth outcomes were additionally adjusted for infant's sex.

† Additionally adjusted for receipt of pertussis vaccine during pregnancy.

‡ Poisson regression model was used because the log binomial model failed to converge for the adjusted model.

§ Reasons for admission: Preterm, Respiratory distress Infection, Feeding problem, Hypoglycaemia, Drug withdrawal, SGA, Birth asphyxia, Congenital abnormality, Phototherapy and Cyanosis.
